# Leflunomide for Connective Tissue Diseases: A Narrative Review of Efficacy and Safety

**DOI:** 10.7759/cureus.104692

**Published:** 2026-03-05

**Authors:** Balakrishnan Navaneethakrishnan, Mahabaleshwar Mamadapur

**Affiliations:** 1 Department of Rheumatology, Avinash Hospitals, Chennai , IND; 2 Department of Clinical Immunology and Rheumatology, JSS Academy of Higher Research, Mysuru, IND

**Keywords:** connective tissue diseases, immunomodulatory therapy, inflammatory arthritis, leflunomide, safety profile

## Abstract

Connective tissue diseases (CTDs) are a heterogeneous group of systemic autoimmune disorders characterized by immune-mediated inflammation and multisystem involvement, frequently requiring long-term immunomodulatory therapy. Leflunomide, a conventional synthetic disease-modifying antirheumatic drug (csDMARD) with established efficacy in rheumatoid arthritis (RA), has increasingly been used off-label in various CTDs, although its clinical role remains incompletely defined. This narrative review synthesizes evidence published between 2015 and 2025 on the efficacy and safety of leflunomide in CTDs, including systemic lupus erythematosus (SLE), systemic sclerosis (SSc), Sjögren’s syndrome, idiopathic inflammatory myopathies (IIMs), mixed connective tissue disease (MCTD), and overlap syndromes.

Available data, largely derived from small clinical trials, observational studies, and case series, indicate that leflunomide may offer clinically meaningful benefit in patients with predominant inflammatory musculoskeletal manifestations, particularly inflammatory arthritis, and may function as a steroid-sparing alternative in individuals who are intolerant of or inadequately responsive to methotrexate. The safety profile of leflunomide in CTD populations appears broadly comparable to that observed in RA when appropriate patient selection, counselling, and laboratory monitoring are employed. However, interpretation of current evidence is limited by heterogeneity in study designs, variable outcome measures, and a lack of disease-specific randomized controlled trials. Leflunomide occupies a defined but limited role as a second-line csDMARD in non-organ-threatening CTDs, highlighting the need for further high-quality studies to clarify its optimal positioning within CTD treatment algorithms.

## Introduction and background

Connective tissue diseases (CTDs) are a heterogeneous group of systemic autoimmune diseases characterised by autoimmunity directed toward connective tissues and immune-mediated inflammation affecting structures such as joints, skin, muscles, vasculature, and internal organs [1]. Although rheumatoid arthritis (RA) has historically been classified within the CTD spectrum, it is intentionally excluded from the analytical scope of this review and is used solely as the established reference indication for leflunomide. This review, therefore, focuses exclusively on systemic lupus erythematosus (SLE), systemic sclerosis (SSc), Sjögren’s syndrome, idiopathic inflammatory myopathies (IIMs), mixed connective tissue disease (MCTD), and overlap syndromes as the target conditions of interest [2]. The CTDs impose a substantial burden due to their chronic, relapsing-remitting, multisystem nature and their significant impact on quality of life and long-term outcomes [3].

Despite their clinical heterogeneity, CTDs share common immunopathogenic pathways, including dysregulation of innate and adaptive immune responses, aberrant activation and proliferation of autoreactive T and B cells, autoantibody formation, and persistent cytokine-driven inflammation [4]. These processes result in progressive tissue damage, organ dysfunction, and, in certain diseases such as SSc, pathological fibrosis [5]. These shared mechanisms form the foundation of immunosuppressive and immunomodulatory treatment strategies, which aim to control disease activity, prevent irreversible damage, and reduce treatment-related morbidity [6].

The therapeutic management of CTDs remains complex and clinically challenging [7]. Glucocorticoids are useful for the rapid control of disease activity but are not used long-term due to cumulative toxicity [8]. The backbone of maintenance therapy consists of conventional synthetic disease-modifying antirheumatic drugs (csDMARDs), including methotrexate, azathioprine, mycophenolate mofetil, cyclophosphamide, and hydroxychloroquine, which function as steroid-sparing agents [9]. Biologic and targeted synthetic disease-modifying antirheumatic drugs (DMARDs) have introduced additional therapeutic options and improved outcomes in selected CTDs [10]. Nevertheless, their use is often limited by high cost, restricted availability, infection risk, and variable efficacy across disease phenotypes [11]. Therefore, there remains a need for efficient, inexpensive, and well-characterised immunomodulatory agents that can be tailored to an individual patient’s clinical profile and are associated with a lower risk of infection [12].

**Figure 1:**
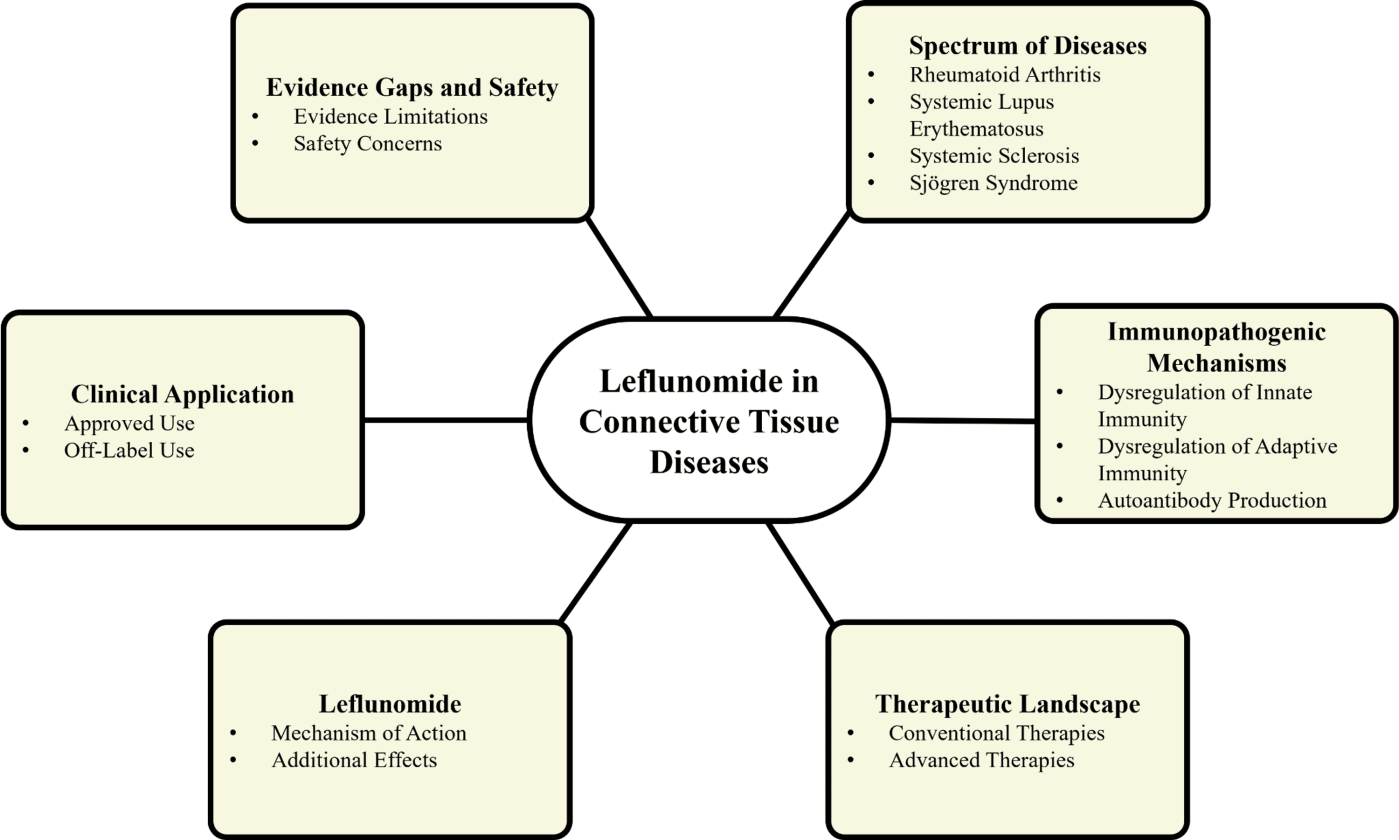
Rationale for leflunomide use in CTDs CTDs: Connective tissue diseases; Flowchart created by authors using Microsoft PowerPoint (Microsoft Corp., Redmond, WA, USA)

Leflunomide is an orally administered immunomodulatory agent with established efficacy in RA [13]. Its active metabolite, teriflunomide, primarily inhibits dihydroorotate dehydrogenase, a key enzyme in the de novo synthesis of pyrimidines [14]. This mechanism selectively suppresses the proliferation of activated T and B lymphocytes, thereby attenuating pathogenic immune responses while relatively sparing resting immune cells [15]. Beyond its antiproliferative effects, leflunomide has been reported to modulate cytokine profiles, reduce autoantibody production, and influence fibroblast activity, mechanisms that are relevant to the immunopathology of multiple CTDs [16]. Figure 1 illustrates the pathophysiological rationale for leflunomide use in CTDs.

Although leflunomide is approved by the United States Food and Drug Administration (US FDA) for the treatment of RA, its pharmacokinetic profile provides a theoretical basis for its potential use in CTDs characterised by inflammatory arthritis, systemic immune activation, and, in selected cases, fibrotic processes [7,17]. Over the past two decades, leflunomide has been used off-label in various CTDs, including SLE, Sjögren’s syndrome, SSc, inflammatory myopathies, and overlap syndromes [18]. Reported benefits include improvement in inflammatory arthritis manifestations, reduction in overall disease activity, and steroid-sparing effects, particularly in patients intolerant of or refractory to methotrexate or other csDMARDs [19]. However, the available evidence is heterogeneous and largely derived from small clinical trials, observational studies, and case series, limiting definitive conclusions [20].

Safety considerations are particularly relevant in CTD populations, where patients frequently present with multisystem involvement and increased susceptibility to infections, cytopenias, and organ dysfunction [21]. Adverse effects, including hepatotoxicity, gastrointestinal intolerance, hypertension, and teratogenicity, necessitate careful patient selection, counselling, and ongoing monitoring [22].

This narrative review critically evaluates the evidence supporting the use of leflunomide across specific CTD phenotypes, with particular emphasis on non-organ-threatening inflammatory musculoskeletal manifestations. It summarises the immunological mechanisms of action relevant to CTDs, assesses available efficacy data across different diseases, and appraises safety and tolerability profiles. Through a disease-by-disease analysis and comparative clinical positioning, the review aims to define the most appropriate phenotype-driven role for leflunomide within contemporary CTD management while identifying key knowledge gaps to guide future research.

Methods

This narrative review was conducted to critically appraise the efficacy and safety of leflunomide across CTDs. A structured literature search was undertaken to inform the narrative synthesis. Electronic databases, including PubMed/MEDLINE, Scopus, Embase, and Google Scholar, were searched for relevant publications from January 2015 to December 2025. The final search was performed in December 2025.

Search strategies combined the term “leflunomide” with disease-specific keywords using Boolean operators (AND/OR), including “connective tissue diseases,” “systemic lupus erythematosus,” “systemic sclerosis,” “Sjögren syndrome,” “idiopathic inflammatory myopathies,” and “mixed connective tissue disease.” Where appropriate, corresponding Medical Subject Headings (MeSH) terms were applied to enhance retrieval sensitivity.

Eligible publications included randomised controlled trials, prospective and retrospective observational studies, and case series reporting clinical use of leflunomide in adult patients with CTDs. Selected review articles were consulted to contextualise findings. Non-human studies, preclinical investigations, editorials, and publications lacking relevant clinical data were excluded. Only articles published in English were considered.

Data were extracted narratively with attention to therapeutic indications, dosing strategies, disease-specific efficacy, organ involvement, and safety outcomes. Findings were synthesised descriptively and organised by disease category to provide a clinically oriented appraisal of leflunomide’s role across connective tissue diseases. Given the narrative design of this review, formal risk-of-bias assessment and PRISMA reporting components were not undertaken.

## Review

Leflunomide pharmacology and mechanism of action

Leflunomide is a csDMARD whose immunomodulatory activity has been well characterised and is mechanistically relevant to the pathogenesis of CTDs [22]. After oral administration, leflunomide is rapidly and almost completely metabolised to its active metabolite, teriflunomide (A77 1726), which mediates its biological effects [23]. Teriflunomide primarily inhibits the mitochondrial enzyme dihydroorotate dehydrogenase (DHODH), a key enzyme in de novo pyrimidine synthesis [24]. De novo pyrimidine synthesis is essential for the proliferation of activated T and B lymphocytes, whereas resting lymphocytes can rely on salvage pathways [25]. Through inhibition of this metabolic pathway, leflunomide selectively suppresses the expansion of activated autoreactive lymphocytes without inducing broad cytotoxic immunosuppression [26]. This mechanism leads to attenuation of pathogenic immune responses, reduction in autoantibody production, and downstream suppression of inflammatory cascades central to CTD pathophysiology [27].

**Figure 2:**
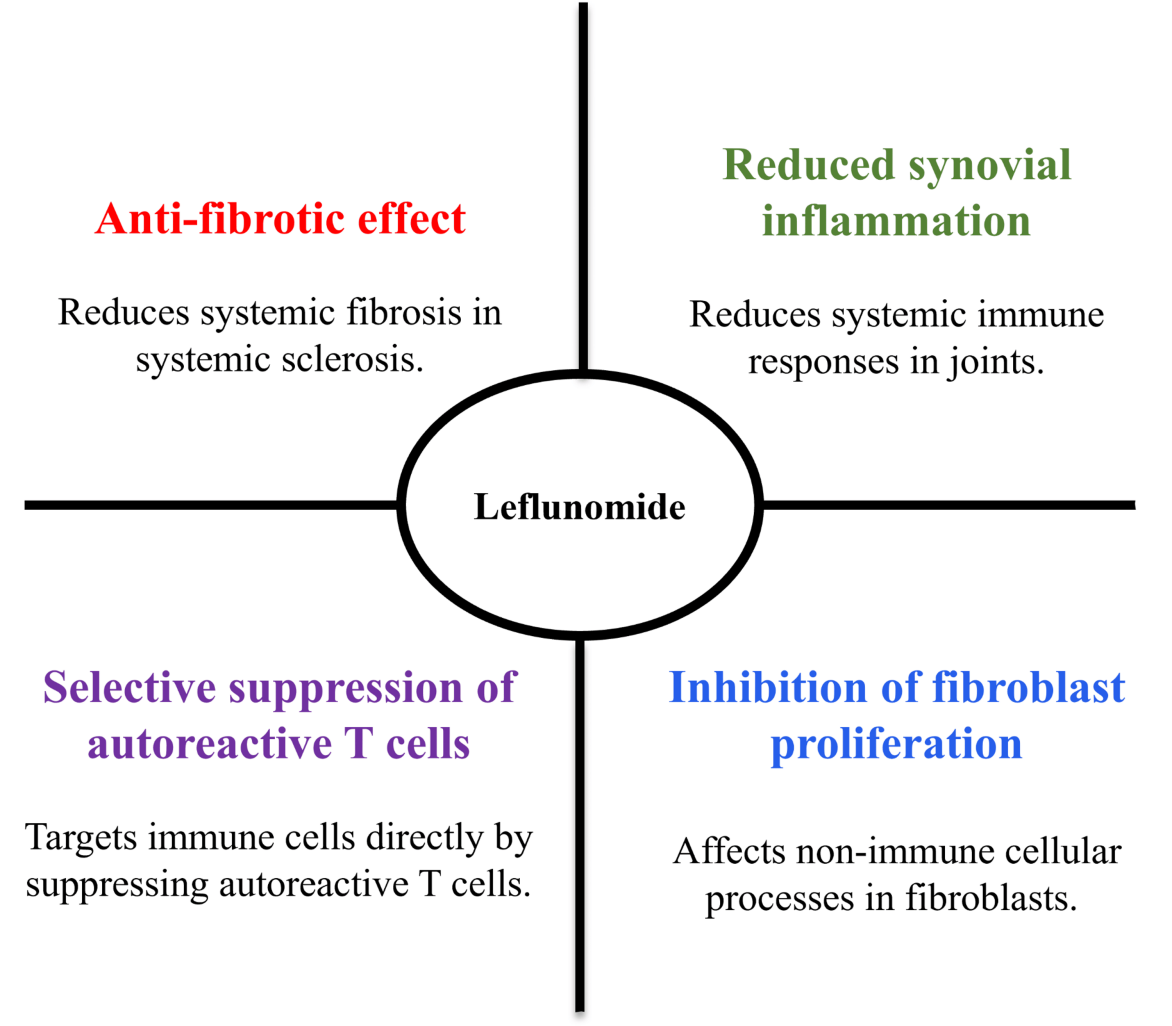
Immunological and cellular effects of leflunomide in CTDs CTDs: Connective tissue diseases; Diagram created by authors using Microsoft PowerPoint (Microsoft Corp., Redmond, WA, USA)

Beyond its antiproliferative effects, leflunomide exerts additional immunomodulatory actions [28]. Experimental studies have demonstrated modulation of pro-inflammatory cytokine production, interference with intracellular tyrosine kinase signalling, and inhibition of nuclear factor kappa B (NF-κB)-mediated transcription [29]. Preclinical investigations have also reported inhibitory effects on fibroblast proliferation, collagen synthesis, and transforming growth factor beta (TGF-β) signalling pathways, suggesting potential anti-fibrotic properties [11,25,26]. These mechanisms contribute to the reduction of synovial inflammation, limitation of immune cell infiltration, and overall suppression of chronic inflammatory activity [30]. Some studies further suggest effects on non-immune cellular pathways relevant to tissue remodelling and fibrosis [24]. Although the clinical relevance of these preclinical findings remains uncertain, they provide a biologically plausible rationale for investigating leflunomide in fibrotic CTDs such as SSc [26]. The pleiotropic immunological and potential anti-fibrotic effects of leflunomide are summarised in Figure 2.

Leflunomide use for RA as a reference framework

Rheumatoid arthritis is the primary disease setting in which leflunomide has been extensively studied, thereby providing an important reference framework for its potential use in other CTDs [28]. Robust evidence from randomised controlled trials and long-term observational studies has established leflunomide as an effective therapy for reducing disease activity, improving functional outcomes, and delaying radiographic progression in RA [29]. Its efficacy is broadly comparable to methotrexate, both as monotherapy and in combination regimens [30]. Practical considerations regarding dosing strategies and safety monitoring have also been informed by clinical experience in RA [22]. The maintenance dose of leflunomide typically ranges around 20 mg/day, whereas loading doses are now used less frequently in clinical practice due to concerns regarding gastrointestinal intolerance and early hepatotoxicity [23]. Experience in RA has also informed recommendations for regular monitoring of liver function tests, haematologic parameters, and blood pressure, guidance that is particularly relevant when extrapolating use to CTD populations [24].

The prolonged half-life of teriflunomide, resulting from extensive enterohepatic recirculation, represents both a pharmacokinetic advantage and a limitation [25]. While sustained drug exposure permits prolonged immunomodulation, it also necessitates specific elimination procedures with cholestyramine in cases of severe toxicity or planned pregnancy [26]. These considerations are especially relevant in CTDs, where multisystem involvement, reproductive considerations, and cumulative exposure to immunosuppression are common [27]. Studies on RA have further demonstrated that leflunomide can function as a steroid-sparing agent and as an alternative in patients intolerant of or contraindicated to methotrexate [28]. Although RA is immunologically and clinically distinct from other CTDs, the extensive evidence base supporting leflunomide in RA provides a cautious framework for anticipating efficacy, toxicity, and long-term monitoring considerations when used off-label in related autoimmune conditions [29].

Leflunomide use for SLE

Systemic lupus erythematosus is a paradigmatic multisystem autoimmune disease characterised by immune complex deposition, autoantibody production, and unpredictable organ involvement [30]. Musculoskeletal manifestations, particularly inflammatory arthritis, are among the most common and persistent features of SLE and are typically managed with long-term immunomodulatory therapy [22]. In this context, leflunomide has primarily been evaluated for lupus-associated arthritis and other non-organ-threatening manifestations [23]. The evidence supporting its use in SLE is derived mainly from small randomised trials, observational studies, and case series [24]. These studies have reported improvements in inflammatory arthritis, including reductions in joint pain, swelling, and functional limitation [25]. Reports of benefit in cutaneous lupus and modest changes in serological disease markers have also been described; however, these findings are based on small cohorts, often involving approximately three to 20 patients, and heterogeneous outcome measures, limiting interpretability [26].

The role of leflunomide in organ-threatening disease, particularly lupus nephritis, remains poorly defined. Available data do not demonstrate efficacy comparable to established induction or maintenance therapies such as cyclophosphamide or mycophenolate mofetil (MMF) [27,28]. Leflunomide should therefore not replace conventional immunosuppressive regimens and is not recommended as either induction or maintenance therapy for lupus nephritis [29]. Its use in this setting may be considered only in carefully selected cases in which standard agents are contraindicated or not tolerated. In contrast, for non-organ-threatening manifestations such as lupus-associated inflammatory arthritis, leflunomide has been explored as an alternative to other arthritis-directed csDMARDs, including methotrexate, rather than as therapy for renal involvement.

Comparative data between leflunomide and other csDMARDs, such as methotrexate or azathioprine, remain limited [27]. Small clinical studies suggest that leflunomide may provide comparable control of inflammatory arthritis, particularly in patients intolerant of methotrexate due to gastrointestinal adverse effects or hepatotoxicity [28]. Its oral administration and potential steroid-sparing effect may make it a practical option in selected patients with chronic musculoskeletal involvement [29]. Safety considerations remain central in SLE, as the disease itself predisposes patients to cytopenias, infections, and organ dysfunction [30]. Reported adverse events in lupus cohorts are broadly consistent with those observed in RA, including gastrointestinal intolerance, elevated liver enzymes, hypertension, and haematologic abnormalities [22]. Serious adverse events appear uncommon under appropriate monitoring; however, strict avoidance during pregnancy and careful laboratory surveillance are essential [23].

Although leflunomide is not recommended as a first-line agent in SLE, the available evidence supports its consideration in carefully selected patients with refractory inflammatory arthritis or intolerance to methotrexate [24]. The absence of large, disease-specific randomised controlled trials underscores the need for further high-quality studies to better define its efficacy, optimal positioning, and long-term safety in SLE [25].

Leflunomide use for SSc

Systemic sclerosis is a heterogeneous CTD characterised by immune dysregulation, vasculopathy, and progressive fibrosis affecting the skin and internal organs [31]. Although fibrosis is a defining feature of SSc, inflammatory manifestations, particularly musculoskeletal involvement, contribute significantly to pain, functional impairment, and reduced quality of life [22]. These features provide a rationale for the selective use of immunomodulatory therapies, including leflunomide, in carefully chosen patients [32]. The use of leflunomide in SSc has largely been confined to patients with prominent inflammatory musculoskeletal involvement, often in the context of overlap syndromes [23]. By inhibiting activated lymphocyte proliferation and modulating cytokine signalling, leflunomide exerts mechanisms similar to those observed in RA, supporting its off-label exploration in SSc-associated inflammatory arthritis [33]. Cases and small observational studies have described improvements in joint pain, swelling, and functional capacity, particularly in patients intolerant of or inadequately responsive to methotrexate [7,13,34].

Preclinical evidence suggesting inhibitory effects on fibroblast proliferation, collagen synthesis, and TGF-β signalling has provided a biological rationale for investigating leflunomide as a potential anti-fibrotic agent; however, these findings have not translated into consistent clinical benefit in SSc [24,25,35]. Clinical reports have not demonstrated sustained improvement in skin thickness scores or internal organ involvement, including interstitial lung disease [2,30,36]. Consequently, current evidence does not support positioning leflunomide as a disease-modifying therapy for fibrosis in SSc [26].

The available evidence base is limited by small sample sizes, often fewer than 20-30 patients, retrospective study designs, and non-controlled trials [27]. Safety considerations are particularly important in SSc, where gastrointestinal, pulmonary, and renal involvement are common [28]. Although reported adverse events appear broadly comparable to those observed in RA, vigilant monitoring remains essential [29]. At present, leflunomide may be considered for inflammatory arthritis in selected patients with SSc, provided that treatment decisions are individualised and guided by careful risk-benefit assessment [30].

Leflunomide use for Sjögren’s syndrome

Sjögren's syndrome is a systemic autoimmune disorder characterised by chronic lymphocytic inflammation of exocrine glands, leading to sicca symptoms and diverse extraglandular manifestations [31]. Inflammatory arthritis is among the most common systemic features and may resemble RA in clinical presentation and immunological profile [22]. This overlap has supported the use of csDMARDs, including leflunomide, in patients with active musculoskeletal involvement [32].

Clinical experience with leflunomide in Sjögren's syndrome has primarily focused on its role in managing inflammatory arthritis and systemic immune activation [33]. Small open-label trials and observational cohorts have reported improvements in joint symptoms, physical function, and inflammatory markers [13]. In this context, leflunomide appears to offer efficacy comparable to other csDMARDs used for inflammatory arthritis, particularly in patients with inadequate responses to hydroxychloroquine monotherapy [35].

Combination therapy represents an area of ongoing investigation in Sjögren syndrome [23]. Pilot studies suggest that leflunomide used in combination with hydroxychloroquine may provide an additive benefit in controlling inflammatory arthritis and selected extraglandular manifestations, including fatigue and serological markers of immune activity [24]. These findings are consistent with a multimodal immunomodulatory approach in a disease characterised by complex and overlapping immune pathways [25]. However, small sample sizes, often fewer than 20 to 30 participants, and short follow-up durations limit the strength and generalisability of these observations [26].

Leflunomide does not appear to significantly improve glandular function or sicca symptoms, reinforcing its role in systemic rather than gland-specific manifestations [27]. Reported adverse events in Sjögren's cohorts are consistent with the established safety profile of leflunomide, with gastrointestinal intolerance and transient elevations in liver enzymes most frequently observed [6,7,28]. Overall, leflunomide may be considered in selected patients with Sjögren's syndrome who have refractory inflammatory arthritis or systemic manifestations, particularly as part of combination therapy; however, adequately powered randomised controlled trials are required to more clearly define its efficacy and safety in this population [29].

Leflunomide use for IIMs

Idiopathic inflammatory myopathies, including polymyositis and dermatomyositis, are characterised by immune-mediated muscle inflammation leading to progressive proximal muscle weakness and functional impairment [30]. Although glucocorticoids remain the cornerstone of therapy, long-term toxicity necessitates the addition of other immunosuppressive agents as steroid-sparing strategies [31]. Leflunomide has been explored in this context, primarily in patients with refractory disease [32].

The evidence supporting leflunomide in IIMs is limited to case reports and small case series [33,34]. These reports describe improvements in muscle strength, reductions in serum creatine kinase levels, and the ability to taper glucocorticoids in selected patients. Leflunomide has most commonly been used as adjunctive therapy following inadequate response or intolerance to conventional agents such as methotrexate or azathioprine [35]. The rationale for its use is based on its capacity to inhibit T-cell-mediated immune responses, which play a central role in muscle inflammation in IIMs [22]. However, clinical responses have been variable, and in the absence of controlled studies, it remains difficult to distinguish true treatment effects from fluctuations in disease activity [23]. Rare cases of myopathy potentially associated with leflunomide have also been reported, warranting caution when prescribing the drug in muscle-predominant disorders [24].

**Figure 3:**
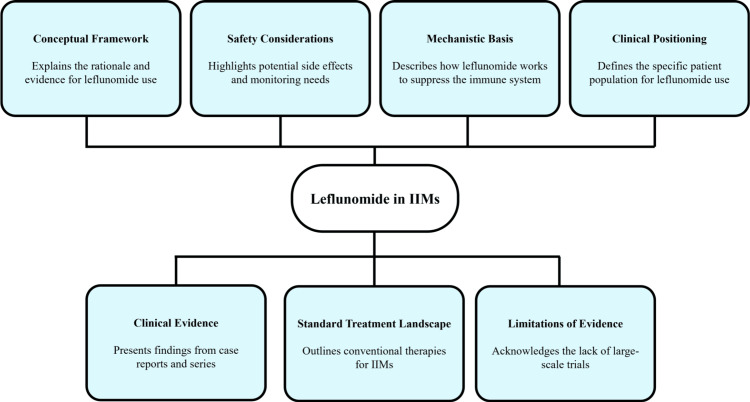
Leflunomide use for IIMs IIMs: Idiopathic inflammatory myopathies; Flowchart created by authors using Microsoft PowerPoint (Microsoft Corp., Redmond, WA, USA)

Given these limitations, leflunomide cannot be recommended as standard therapy for IIMs [25]. Its use may be considered only in carefully selected cases, with close monitoring of muscle enzymes, liver function tests, and clinical status [26]. Further controlled clinical trials are required to clarify its efficacy, safety, and positioning within treatment algorithms for inflammatory myopathies [27]. Figure 3 summarises the rationale, available evidence, and clinical positioning of leflunomide in IIMs.

Leflunomide use for MCTD and overlap syndromes

Overlap syndromes and MCTDs comprise a heterogeneous group of clinically and immunologically related CTDs characterised by the coexistence of features from two or more distinct CTDs, most commonly SLE, SSc, and IIMs [36]. Inflammatory arthritis is a frequent and often chronic manifestation, contributing substantially to functional impairment and long-term morbidity [22]. The phenotypic diversity of overlap syndromes complicates therapeutic decision-making and necessitates an individualised, phenotype-driven treatment approach [37].

The use of leflunomide in MCTD and overlap syndromes has primarily focused on inflammatory musculoskeletal manifestations, particularly when arthritis resembles RA in pattern and chronicity [38]. Its application is extrapolated from established efficacy in RA and its immunomodulatory effects on activated T and B lymphocytes [39]. In clinical practice, leflunomide may be considered when hydroxychloroquine and low-dose glucocorticoids provide inadequate control, or when methotrexate is not tolerated or contraindicated [40].

Evidence supporting leflunomide in overlapping CTDs remains limited to small case series and anecdotal reports [41]. These reports describe improvements in joint pain, reductions in inflammatory biomarkers, and steroid-sparing effects in selected patients [42]. However, its impact on non-articular manifestations, including cutaneous lupus, myositis, or internal organ involvement, has not been systematically evaluated [43]. Furthermore, the absence of standardised outcome measures and controlled study designs precludes definitive conclusions regarding efficacy [44].

Given these limitations, the use of leflunomide in MCTD and overlap syndromes remains empirical and should be guided primarily by the dominant clinical phenotype rather than by diagnostic categorisation alone [45]. Careful assessment of organ involvement, prior treatment exposure, and cumulative immunosuppressive burden is essential when considering therapy [46]. Although leflunomide may represent a reasonable option when inflammatory arthritis predominates, its effect on overall disease trajectory in overlap CTDs remains insufficiently studied and warrants further investigation [47].

Safety profile of leflunomide in CTDs

Safety considerations are particularly important when leflunomide is used in CTDs, as patients frequently have multisystem involvement and may receive concomitant immunosuppressive therapies [48]. Overall, the safety profile of leflunomide in CTD populations appears broadly comparable to that observed in RA; however, disease-specific factors may influence susceptibility to adverse effects and overall tolerability [49]. The most frequently reported adverse events include gastrointestinal symptoms such as diarrhoea, nausea, and abdominal discomfort, which are generally dose-dependent [22]. Hepatotoxicity, manifested by elevations in liver transaminases, represents a clinically significant concern and may be exacerbated by concomitant hepatotoxic medications or underlying liver disease [50]. Regular monitoring of liver function tests is therefore recommended, particularly during the initial months of therapy and following dose escalation [23].

Other commonly reported adverse effects include alopecia, hypertension, and weight loss [24]. New-onset or worsening hypertension has been described and necessitates routine blood pressure monitoring [25]. Alopecia is typically mild and reversible but may negatively affect treatment adherence [26]. Haematologic abnormalities, including leukopenia and anaemia, occur less frequently but may carry particular relevance in CTD populations predisposed to cytopenias due to underlying disease activity or concomitant therapies [27]. Infection risk is another important consideration, especially in patients receiving combination immunosuppressive regimens [28]. While leflunomide monotherapy has not consistently been associated with a marked increase in severe infections, the cumulative immunosuppressive burden from glucocorticoids and other agents may heighten susceptibility [29]. Close clinical surveillance and prompt evaluation of infectious symptoms are therefore essential [30].

Teratogenicity represents a major limitation of leflunomide use, particularly in women of childbearing potential [31]. Leflunomide is contraindicated during pregnancy, and effective contraception is required for both female and male patients during treatment [32]. In cases of planned conception or unintended exposure, accelerated drug elimination using cholestyramine is recommended [33]. These considerations are especially relevant in CTDs, which predominantly affect women of reproductive age [34]. When prescribed with appropriate patient selection, counselling, and systematic monitoring, leflunomide is generally well tolerated in CTD populations [35]. However, its prolonged half-life and potential for delayed toxicity underscore the importance of proactive safety monitoring [36]. Key safety considerations and monitoring requirements for leflunomide use in the treatment of CTDs are summarised in Table 1.

**Table 1:** Safety profile of leflunomide in CTDs CTD: Connective tissue disease, RA: Rheumatoid arthritis

Safety domain	Adverse effects	Clinical implications	Management	Reference no.
Safety context	Multisystem disease and concomitant immunosuppression	Increased vulnerability to adverse events	Individualized risk–benefit assessment	[48]
Disease-specific tolerability	Comparable to RA with CTD-specific modifiers	Variable susceptibility across CTDs	Disease- and phenotype-based monitoring	[49]
Gastrointestinal toxicity	Diarrhoea, nausea, abdominal discomfort (dose-dependent)	May affect adherence and tolerability	Dose adjustment, symptomatic management	[22]
Hepatotoxicity	Elevated liver transaminases	Risk increased with hepatotoxic drugs or liver disease	Regular liver function tests	[50]
Blood pressure effects	New-onset or worsening hypertension	Cardiovascular risk	Routine blood pressure monitoring	[25]
Alopecia and weight loss	Usually mild and reversible	May affect patient compliance	Patient counselling and reassurance	[26]
Hematologic toxicity	Leukopenia, anaemia	Higher risk in CTDs with baseline cytopenias	Regular complete blood counts	[27]
Infectious risk	Increased with combination immunosuppression	Potential for serious infections	Vigilant clinical surveillance	[28]
Monotherapy infection risk	No major increase in severe infections	Safer when used alone	Cautious combination therapy	[29]
Teratogenicity	Contraindicated in pregnancy	Major reproductive safety concern	Strict contraception	[31]
Drug elimination	Prolonged half-life due to enterohepatic circulation	Delayed toxicity possible	Cholestyramine washout if needed	[33]
Tolerability	Generally well tolerated with monitoring	Safe in selected patients	Proactive safety management	[36]

Comparative effectiveness and positioning in CTD treatment algorithms

Careful consideration is required when comparing the positioning of leflunomide within treatment algorithms for CTDs against other conventional immunosuppressive agents such as methotrexate, azathioprine, and MMF [37]. Differences in mechanism of action, organ specificity, safety profile, and supporting evidence base influence therapeutic selection [38]. In many CTDs with prominent inflammatory arthritis, methotrexate is commonly used as a first-line csDMARD due to its extensive evidence base and clinical familiarity [39]. However, intolerance and contraindications, particularly hepatic dysfunction or inadequate response, may necessitate alternative therapies [40]. In such situations, leflunomide represents a mechanistically distinct option with comparable efficacy in inflammatory joint disease, particularly in RA-like phenotypes [41].

Systemic or organ-threatening manifestations, including lupus nephritis, interstitial lung disease, and inflammatory myopathies, are more commonly managed with agents such as azathioprine or MMF [42]. In contrast, leflunomide lacks robust evidence in these settings and should not be considered a substitute for therapies with established organ-specific efficacy [43]. Its most clearly defined role remains in non-organ-threatening diseases, particularly musculoskeletal involvement [44]. Practical considerations also influence therapeutic positioning [45]. Leflunomide is administered orally and is generally available as a lower-cost generic agent, which may increase its appeal in resource-limited settings compared with biologic or targeted synthetic therapies [46]. When used as monotherapy or in combination with hydroxychloroquine, it may contribute to improved control of inflammatory arthritis and facilitate glucocorticoid dose reduction in selected patients [47]. Nonetheless, combination regimens involving hepatotoxic or immunosuppressive agents require close monitoring [48].

Leflunomide therefore occupies a defined but limited niche within CTD treatment strategies as a second-line or alternative csDMARD for patients with inflammatory arthritis who are intolerant of or inadequately responsive to methotrexate [49]. Its role in systemic or organ-threatening disease remains unestablished, and further disease-specific studies are required to clarify its optimal positioning across the spectrum of CTDs [50]. The comparative positioning of leflunomide within CTD treatment algorithms is summarised in Table 2.

**Table 2:** Positioning of leflunomide among csDMARDs in CTDs CTDs: Connective tissue diseases, csDMARD: Conventional synthetic disease-modifying antirheumatic drug, DHODH: Dihydroorotate dehydrogenase, MMF: Mycophenolate mofetil

Drug	Mechanism of action	Primary clinical role in CTDs	Efficacy on organ-threatening disease	Key limitations	Reference no.
Methotrexate	Antimetabolite inhibiting folate-dependent pathways	First-line csDMARD for inflammatory arthritis in CTDs	Limited	Intolerance, hepatotoxicity, contraindicated in liver disease	[9]
Leflunomide	DHODH inhibition → suppression of activated T and B lymphocytes	Second-line or alternative csDMARD for inflammatory arthritis	Not established	Teratogenicity, hepatotoxicity, and limited organ efficacy	[41]
Azathioprine	Purine synthesis inhibition	Systemic and organ-threatening CTD manifestations	Established (e.g., lupus nephritis)	Myelotoxicity, infection risk	[27]
Mycophenolate mofetil	Inhibition of inosine monophosphate dehydrogenase	Organ-threatening disease (renal, pulmonary, muscular)	Established	Gastrointestinal intolerance, infection risk	[12]
Leflunomide (comparative)	Mechanistically distinct from methotrexate	Alternative to methotrexate intolerance	Inferior to azathioprine/MMF	Insufficient evidence in severe disease	[3]
Leflunomide (practical considerations)	Oral administration, low cost	Suitable for resource-limited settings	Limited	Requires close monitoring in combinations	[16]
Leflunomide + hydroxychloroquine	Complementary immunomodulation	Additive benefit in inflammatory arthritis	Not indicated	Hepatic and immunosuppressive overlap	[27]
Overall positioning	Second-line csDMARD	Non–organ-threatening inflammatory arthritis	Not recommended	Evidence gaps in severe CTDs	[49]

Limitations and future directions

Various methodological and clinical limitations restrict the interpretation of the current evidence regarding the use of leflunomide in CTDs. Most available data are derived from small observational studies, uncontrolled trials, and case reports, limiting the strength and external validity of the findings. Considerable heterogeneity in study design, patient populations, disease phenotypes, and outcome measures further complicates direct comparisons across studies. Selective reporting and publication bias also cannot be excluded. Much of the clinical rationale for leflunomide use in CTDs is extrapolated from RA, which may not adequately reflect the distinct immunopathology, organ involvement, and disease trajectory of other CTDs.

Future research should consist of well-designed, adequately powered randomised controlled trials targeting specific CTDs and clearly defined clinical phenotypes. Standardised outcome measures encompassing inflammatory, functional, and systemic domains should be implemented to enhance comparability and clinical relevance. Long-term, disease-specific safety registries are necessary to better characterise cumulative toxicity and infection risk. Vigilance for rare but severe complications is crucial during any long-term systemic therapy, as documented in a previous report, underscoring the need for sustained monitoring [51]. Research into predictive biomarkers of treatment response and rational combination strategies, particularly in steroid-sparing contexts, may facilitate more precise positioning of leflunomide within CTD treatment algorithms. In addition, given that many applications in CTDs remain off-label, considerations related to regulatory approval status, reimbursement policies, and local prescribing frameworks are relevant, as access may vary across jurisdictions despite leflunomide generally being available as a lower-cost generic agent.

## Conclusions

Leflunomide is an effective immunomodulatory agent in the therapeutic arsenal of the treatment of CTDs, especially in patients whose musculoskeletal manifestations are mostly inflammatory in nature. Its clear mechanism of action, oral route of administration, and long history of CTDs in RA give it a solid basis for proceeding with conservative off-label administration in a few CTDs. The reviewed evidence in this article indicates that leflunomide may provide significant clinical value in conditions such as SLE, Sjögren’s syndrome, SSc-related arthritis, overlap syndromes, and refractory inflammatory myopathy, particularly in those intolerant or poorly responsive to methotrexate. Notably, its safety profile in CTD populations seems to be largely similar to that seen in RA, in the case of using careful patient selection, counselling, and follow-up. However, the existing body of evidence is limited by small research and varying results, creating a need to conduct new, high-quality research. Until more robust data are available, leflunomide is best positioned as an arthritis-directed second-line csDMARD for non-organ-threatening CTD, particularly in patients with predominant inflammatory musculoskeletal involvement. Its use should be individualised according to disease phenotype, comorbidities, and overall risk-benefit assessment.
